# BIOABSORBABLE CAGES IN SPINAL FUSION IN AN ANIMAL MODEL: A SYSTEMATIC REVIEW AND META-ANALYSIS

**DOI:** 10.1590/1413-785220253306e294038

**Published:** 2025-11-10

**Authors:** Sylvio Mistro, Marcelo Italo Risso, Rafael Magalhães Grana, Mauricio Coelho Lima, André Frazão Rosa, Alberto Cliquet

**Affiliations:** 1 Universidade Estadual de Campinas (UNICAMP), Departamento de Ortopedia Reumatologia e Traumatologia, Campinas, Sao Paulo, SP, Brazil.

**Keywords:** Arthrodesis, Spinal Fusion, Absorbable Implants, Meta-Analysis, Models, Animal, Artrodese, Fusão Intervertebral, Implantes Absorvíveis, Meta-Análise, Modelo Animal

## Abstract

To evaluate the efficacy of bioabsorbable interbody cages in comparison with conventional techniques in animal models, with emphasis on the impact of follow-up time on developments. A systematic review and meta-analysis was performed including 11 studies on the use of bioabsorbable cages in comparison with conventional techniques. The odds ratio (OR) was calculated for range of motion (ROM), and heterogeneity was assessed by Cochran's Q test. Descriptive statistical analyses and hypothesis tests were performed to evaluate the parameters of fusion rate, intervertebral disc height and ROM. The 11 studies included totaled 244 animals. The analysis revealed a cumulative OR of 1.70 for ROM and fusion rate in the first four months of follow-up. No significant differences were found in height parameters in the study follow-ups. Heterogeneity among studies was low, indicating consistency in the results. The analysis suggests that bioabsorbable cages have advantages in periods of less than four months, and that there is no inferiority in the results in follow-up periods longer than four months in terms of fusion rate, ROM and intervertebral height in long-term experimental studies, and further research is needed to determine their clinical applicability. ***Level of Evidence ll; Systematic meta-analytical review of non-randomized controlled clinical studies whose results were homogeneous***.

## RESUMO

Avaliar a eficácia através do desempenho biomecânico e clínico dos dispositivos intersomáticos bioabsorvíveis na fusão intervertebral em comparação com técnicas convencionais em modelos animais, com ênfase no impacto do tempo de acompanhamento sobre os desfechos. Realizou-se uma revisão sistemática e metanálise com a inclusão de 11 estudos sobre o uso de dispositivos bioabsorvíveis em fusão intersomática vertebral, em comparação com os implantes mais comumente usados na rotina da cirurgia de coluna. A Odds Ratio (OR) foi calculada para a amplitude do movimento (ROM), e a heterogeneidade foi avaliada pelo teste Q de Cochran. Análises estatísticas descritivas e testes de hipóteses foram realizados para avaliar os parâmetros taxa de fusão, altura discal intervertebral e ROM. Os 11 estudos incluídos somaram 244 animais. A análise revelou uma OR acumulada de 1,70 para ROM e taxa de fusão, nos primeiros quatro meses de seguimento. Não foram encontradas diferenças significativas nos parâmetros de altura nos seguimentos dos estudos. A heterogeneidade entre os estudos foi baixa, indicando consistência nos resultados. A análise sugere que os cages bioabsorvíveis apresentam vantagens em períodos de acompanhamento inferiores a quatro meses, e que não há inferioridade nos resultados em períodos de acompanhamento maiores que quatro meses em termos de taxa de fusão, identificadas pela ROM e altura intervertebral. ***Nível de Evidência ll: Revisão sistemática metanalítica de estudos clínicos controlados não randomizados cujos resultados foram homogêneos***.

## INTRODUCTION

The treatment of spinal pathologies with interbody fusion (arthrodesis), with or without decompression, plays a crucial and well-established role in degenerative, traumatic, infectious, and tumor conditions.^1-4.^

The first reports of spinal arthrodesis date back to the early 20th century, in studies by Fred Albee,^5^ in which arthrodesis was achieved after slices of tibia were positioned and sutured between spinous processes, and by Russel Hibbs,^6^ in which fusion was achieved without the use of grafts, through osteotomy and subsequent approximation of the spinous process to the vertebral lamina.^6,7^ In 1933, Burns^8^ performed the anterior interbody fusion procedure for the treatment of lumbar spondylolisthesis using a structured tibial graft, and later, in the 1950s, Hodgson^9^ and Stock^10^ described interbody arthrodesis via anterior access for the treatment of tuberculosis.

In the cervical spine, anterior arthrodesis with discectomy became the gold standard for the treatment of several pathologies, including degenerative disc disease, myelopathies, and traumatic injuries. This technique has evolved since the descriptions by Cloward^11^ and Smith and Robinson^12^ in the 1950s.

Advances in fixation techniques followed with the development of Luque's sublaminar wiring^13^ and later with Judet^14^ e and Roy-Camille et al.,^15^ who elaborated and disseminated spinal fixation through pedicle screws. The evolution of transpedicular screw fixation continued with more versatile and robust systems applicable to the treatment of deformities, trauma, and degenerative conditions, with historical highlights including Margerl's internal fixation system^16^ and Cotrel et al.'s deformity system.^17^

In parallel with the development of fixation implants, interbody devices—or "cages"—were also introduced.^18-20^

The use of these devices has become very common in spinal fusion surgery, consisting of implanting a support in the intervertebral space with an inner cavity to be filled with graft material.^21^

The initial presentation was by Bagby^22^ in 1988, who, in studies on horses with cervical spondylotic myelopathy, developed a cylindrical steel interbody device with an internal space to be filled with graft material, which was then press-fit into the intervertebral space—known as the "Bagby Bone Basket." In the early 1990s, the technique was expanded and developed in humans, for both cervical and lumbar spine surgeries.^22,23.^

These devices possess mechanical properties to withstand compressive loads, provide a large surface area for bone graft placement to promote fusion, and improve biomechanical stability. They may also serve as a vehicle for the local delivery of medication to the surgical site.^2,19,24,25^

Among the available devices, those made of titanium or PEEK (polyether-ether-ketone) are the most commonly used in spinal surgery today.^26,27^

It is well known that interbody devices made of metal, carbon fiber, or PEEK are non-absorbable materials, which do not allow for complete biological fusion and remain as foreign bodies in the host organism. This can lead to foreign body reactions and, not infrequently, the need for revision surgeries, as well as implant breakage, migration, subsidence, and other complications.^22,28^

Research on the development of an interbody device capable of overcoming or minimizing the undesirable outcomes of conventional implants has always been a focus.^19^

Such a device should provide adequate intervertebral support, demonstrate appropriate biocompatibility and properties as close as possible to host bone, be highly permeable to imaging studies, replace the intervertebral disc or affected area, and promote proper fusion through the concomitant use of bone grafts.^19,22^

In this context, a potential solution is an interbody device manufactured from bioabsorbable material, which, in addition to the aforementioned properties, would have the advantage of being gradually reabsorbed by the organism in a controlled manner.^19,22,29^

The most commonly used bioabsorbable materials are polymers such as polylactic acid (PLA), poly-L-lactic acid (PLLA), polyglycolic acid (PGA), and poly-D,L-lactic-co-glycolic acid (PLGA),^30,31^ ceramics such as hydroxyapatite (HA), tricalcium phosphate (TCP), beta-tricalcium phosphate (β-TCP), calcium sulfate, and bioactive glass,^32^ as well as magnesium (Mg) and its alloys.^33^

The primary goal of interbody arthrodesis is to eliminate motion at the operated segment, and its progress can be assessed through imaging studies.^4^ Outcomes may be evaluated by measuring fusion rate, range of motion (ROM) of the segment, and intervertebral disc height.

The present study aims to conduct a systematic review and meta-analysis to assess the performance of bioabsorbable interbody devices in animal models of interbody fusion, evaluating fusion rate, intervertebral disc height, and ROM, in comparison with the most commonly employed implants in routine spine surgery, including PEEK, titanium, and structured tricortical bone grafts.

## METHODOLOGY

A systematic review of the literature was conducted as a secondary study, following the Cochrane Handbook for Systematic Reviews of Interventions (version 6.1, 2020) and PRISMA (Preferred Reporting Items for Systematic Reviews and Meta-Analyses) guidelines. Studies were identified through systematic searches in electronic databases and research portals, in addition to reference list analyses, using 10 databases and keywords based on MeSH descriptors and free terms: *Arthrodesis* AND ("*spine*" OR "*spine fusion*" OR "*spinal fusion*") AND (*cage* OR *interbody device* AND ("*absorbable implants*" OR (*materials* OR *Material* AND (*absorbable* OR *bioabsorbale* OR *biodegradable*) OR *"biodegradable* cage").

Inclusion criteria comprised studies conducted in ovine or caprine animal models, including randomized or non-randomized clinical trials, controlled observational studies, or case series, with no restrictions on year or language. Exclusion criteria included human or in vitro studies, literature reviews, case reports, interviews, commentaries, duplicate articles, and those that did not evaluate arthrodesis outcomes in the cervical or lumbar spine, or were not published in full, even after attempts to contact the authors for data retrieval. Outcomes assessed included fusion rate, segmental range of motion (ROM), and intervertebral disc height.

**Figure 1:**
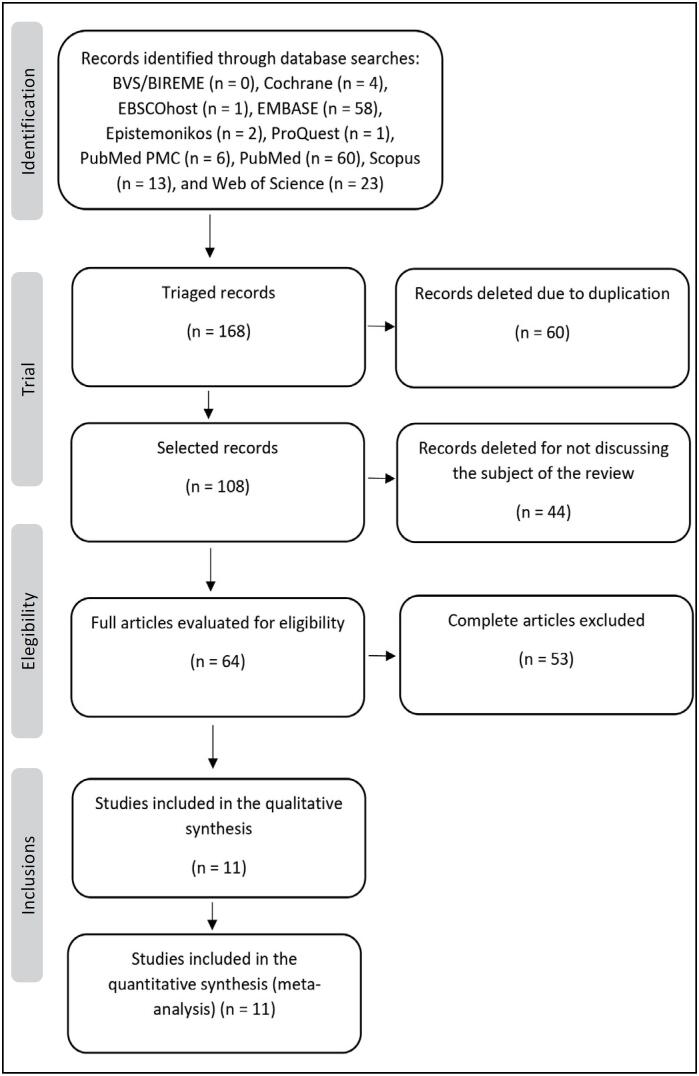
Flowchart of the process of identification and exclusion of articles.

The systematic search identified 168 articles, distributed across databases as follows: BVS/BIREME (n = 0), Cochrane (n = 4), EBSCOhost (n = 1), EMBASE (n = 58), Epistemonikos (n = 2), ProQuest (n = 1), PubMed PMC (n = 6), PubMed (n = 60), Scopus (n = 13), and Web of Science (n = 23). These articles were exported to the reference management programs EndNote and Rayyan, including titles, abstracts, references, and data sources. Duplicate studies were automatically removed, resulting in the exclusion of 60 articles and leaving 108 for eligibility screening. After full-text evaluation, 64 articles were reviewed, and 11 were considered relevant and selected for data extraction, as shown in Figure 1.

Data extraction began with the independent assessment of titles and abstracts from the identified studies by two researchers. Full texts of potentially relevant studies were subsequently retrieved and independently reviewed by the same researchers. Each researcher compiled a list of studies considered to meet the predefined inclusion and exclusion criteria, using a standardized clinical form to record exclusion reasons and document the article selection flow. Lists were compared, and disagreements were resolved by discussion and consensus; if consensus was not reached, the article was assessed by a third independent reviewer for final inclusion. Evidence quality and strength of recommendations were assessed using the Newcastle-Ottawa Scale (NOS) for non-randomized methodology studies, case-control, and cohort designs. This scale evaluates study quality based on a star system, analyzing study group selection, comparability, and assessment of exposure or outcome of interest.

Fusion rate and ROM were evaluated using the approach proposed by Cahill et al.^34^ while disc height was calculated using the mean of anterior, middle, and posterior intervertebral space measurements. The main endpoint consisted of data analysis obtained from animal models (ovine or caprine), according to variables observed in case-control studies. The Odds Ratio (OR) was calculated for ROM, based on reported results in the selected articles. In addition to association measures, the Mantel-Haenszel fixed-effect model^35^ was applied to assess analytic outcomes. These models consider the following calculation: Yj (desired effect) = θM + εj (where εj is the random error of the study, and θM is the common effect across all studies).

Heterogeneity was examined using Cochran's Q test, in which the null hypothesis states that studies included in the meta-analysis do not present heterogeneity in relation to randomized analyses and therapeutic interventions. Effectiveness rates of surgeries, obtained from hypothesis tests and association measures reported in the included articles, were analyzed in paired groups of animals treated and untreated with bioabsorbable interbody devices, according to the I^2^ index described by Thompson and Higgins.^36^ According to the authors, I^2^ = (Q − df) / Q × 100, where Q is based on Cochran's Q test (Q = Σwi (θi − θ)^2^), which tests the null hypothesis that the included studies are homogeneous. Descriptive statistical analyses of the selected articles were performed, with hypothesis tests adopting a significance level of 0.05. All statistical analyses were conducted using JASP software, version 0.19.2 (2024).

Each of the 11 selected studies was assessed based on epidemiological methodology, risk of sampling bias, applied statistics, and probabilistic/statistical inference using hypothesis tests (Student's t-test or Mann-Whitney test). The null hypothesis (H_0_) of this study was defined as a statistical parameter whereby the studies should be heterogeneous, based on mean values greater than 50% between Mantel-Haenszel and I^2^ tests, calculated over the geometric mean and variance of the statistical outcomes of the 11 studies.

Analyses related to disc height were assessed using Pearson's correlation coefficient and Student's t-test, based on time variables and the use of bioabsorbable interbody devices. Variables such as ROM and fusion were evaluated together with consolidation time, with mean differences and confidence intervals assessed by hypothesis testing based on data parametricity (Student's t-test), using an alpha level of 0.05.

## RESULTS

After applying the exclusion criteria, 11 articles were included in the statistical analyses, considering the description of methodologies applied in paired case-control trials (cases treated with bioabsorbable interbody devices and controls treated with conventional techniques—in these studies, bone grafting, PEEK interbody devices, and titanium interbody devices).

A Figure 1 shows the selection process of the studies that composed the final sample of this review.

The selected studies showed low heterogeneity when pooled, as described in Table 1.

**Table 1:** Q test and I^2^ test values for the analysis of studies that evaluated fusion outcomes, ROM, and disc height, comparing bioabsorbable devices and conventional techniques between 2002 and 2024.

	Q test	Value of p	I^2^
Coefficient	< 0.05	< 0.05	28%

Regarding the analytical factors involving performance, we obtained a pooled OR of 1.70 (CI 1.27–2.04) for biomechanical analyses (ROM) and fusion rate up to 4 months of postoperative follow-up, as presented in Table 2.

**Table 2:** Measures of association (OR) and respective confidence intervals of the studies analyzed, including sample data for ROM and fusion rate up to 4 months of follow-up.

Parameters	Number of studies	Number of eligible animals in studies	Mean of OR to ROM* and fusion rate (compared with conventional treatment)
Pooled results	11	244	1.70 (1.27-2.04)
Date of analysis			
Between 2002-2004	4 (36.3%)	38 (16.0%)	1.52 (1.01- 2.01)
Between 2004-2024**	7 (64.7%)	206 (84.0%)	1.89 (1.21- 2.43)

CI = Confidence Interval (95%).

* Values based on flexion, extension, and rotation.

** Larger sample size and 20-year publication interval between 2004 and 2024.

In total, the 11 selected studies included 280 animals (mean of 31), ranging from 10 to 45 subjects. A total of 36 animals were excluded from the statistical calculations, leaving 244 animals, due to follow-up periods shorter than 4 months or longer than 12 months, as well as cases in which animals were retested at different time points, in order to minimize sampling bias.

The data collection characteristics of each evaluated study are described in Table 3.

**Table 3:** Data collection and results of hypothesis tests for the difference between incidence rates according to follow-up time for fusion rate and ROM.

	nº of animals*	Data source	Bioabsorbable material**	Fusion rate comparison:*** (p)	ROM comparison**** (p)
Toth et al. (2002)^37^	10	Milwaukee, EUA	70-30 D, L-PLA	< 0.05	< 0.05
Wuisman et al. (2002)^38^	36	Amsterdam, Holanda	PLLA	< 0.05	< 0.05
Cahill et al. (2003)^34^	12	Tampa, EUA	PLA-PGA	< 0.05	< 0.05
Kandziora et al. (2004)^39^	24	Berlin, Germany	PLLA	< 0.05	< 0.05
Daentzer et al. (2014)^29^	24	Hannover, Germany	Mg-PCL	< 0.05	< 0.05
Li et al. (2014)^40^	24	Xi'an Shaanxi, China	PCL-TCP	< 0.05	< 0.05
Li et al. (2015)^41^	24	Shanghai, China	PDLLA	< 0.05	< 0.05
Cao et al. (2017)^28^	18	Shanghai, China	PLA-TCP	< 0.05	< 0.05
Ren et al. (2017)^42^	24	Lianyungang, China.	MAACP-TCP	< 0.05	< 0.05
Xu et al. (2018)^43^	24	Shanghai, China	Mg-Zn	< 0.05	< 0.05
Yang et al (2024)^44^	24	Shanghai, China	Mg-Zn-Nd-Zr	< 0.05	< 0.05

* In the studies addressed, animals considered outliers with respect to time, as well as those evaluated at two different time points, were excluded.

** 70-30 D,L-PLA (poly-D-lactic acid), PLLA (poly-L-lactic acid), PLA-PGA (polylactic–polyglycolic acid), Mg-PCL (magnesium–polycaprolactone), PCL-TCP (tricalcium phosphate–polycaprolactone), PDLLA (poly-D,L-lactic acid), PLA-TCP (polylactic acid–tricalcium phosphate), MAACP-TCP (multiamino acid–tricalcium phosphate), Mg-Zn (magnesium–zinc), Mg-Zn-Nd-Zr (magnesium–zinc–neodymium–zirconium).

*** Fusion rate comparison: Bioabsorbable devices vs. conventional devices < 4 months of observation (p-values obtained by Student's t-test).

**** ROM comparison: Bioabsorbable devices vs. conventional devices < 4 months of observation. p-values obtained by Student's t-test based on flexion, extension, and rotation.

**Figure 2:**
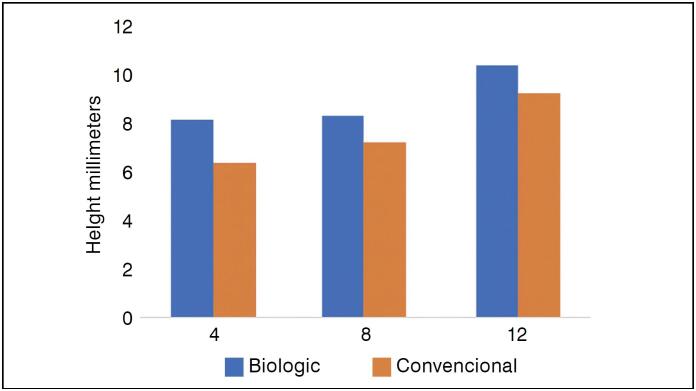
Height in millimeters, compared across 3 time points based on means and 95% CIs, as observed in the 6 studies analyzed using Pearson's regression technique* (R^2^ = 0.96), p > 0.05 (Student's t-test) *Mean of 8.6 mm in cages, with a maximum reach of 10 mm at 12 months.

The follow-up time of the groups (mean of eight months) did not show significant differences regarding fusion rates and ROM, based on Student's t-test results (p > 0.05) at time points greater than 4 months. However, at time points shorter than four months, bioabsorbable devices showed better performance in fusion rates and ROM, also based on Student's t-test (p < 0.05), as shown in Table 2. Over the total follow-up period, no significant difference was observed in the final intervertebral disc height, as shown in Figure 2.

**Figure 3:**
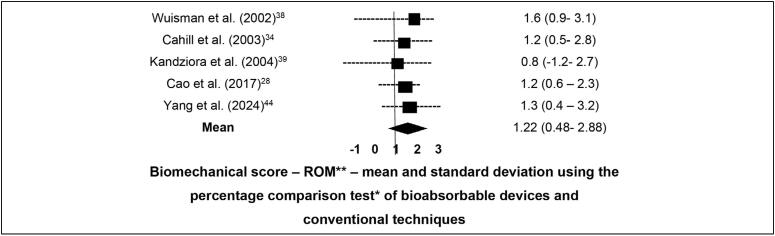
Forest plot comparing ROM between bioabsorbable devices and conventional techniques at follow-up > 4 months.

**Figure 4:**
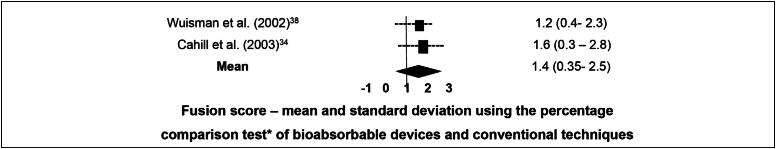
Forest plot comparing fusion rates between bioabsorbable devices and conventional techniques at follow-up > 4 months.

Figure 3 presents the Forest Plot combining the studies that performed direct comparisons of ORs, means, and standard deviations for ROM, and Figure 4 shows the same for fusion rate, based on studies with paired and controlled groups. These comparisons between bioabsorbable interbody devices and conventional techniques did not demonstrate significant improvement at follow-up longer than 4 months.

** Score based on flexion–extension movement as proposed by Cahill et al.^34^

*Histomorphometric analysis based on the percentage of newly formed trabecular bone, following the study by Cahill et al.^34^

Figure 4 shows the assessment of intervertebral disc height, comparing bioabsorbable interbody devices with conventional devices. This parameter showed no significant difference throughout the entire follow-up period, with p > 0.05 (Student's t-test) and R^2^ = 0.96.

## DISCUSSION

The present systematic review and meta-analysis aimed to evaluate the efficacy of intervertebral fusion techniques in animals using bioabsorbable devices compared with the cages most commonly employed in spine surgery practice, namely structured autologous bone alone, PEEK, and metals, particularly titanium. Studies conducted in sheep and goats are relevant because the size of the vertebrae and the lamellar bone growth rate in these animals are comparable to humans, which allows human surgical techniques and instrumentation to be applied with ease.^45,46^

The results of this study demonstrated that bioabsorbable interbody devices performed better with respect to fusion rates during periods shorter than four months of follow-up; after this period, the differences between groups were not significant. In the study by Xu et al.^43^ it was shown that, for magnesium interbody devices in sheep subjected to cervical arthrodesis, no significant results were found for fusion rates after 6 months of postoperative evaluation. Similar findings were reported by Kandziora et al.^20^ who also did not demonstrate improved fusion rates with cervical interbody devices at 3 months of follow-up. On the other hand, Lippman et al.^30^ observed adequate fusion rates at 6 months of cervical arthrodesis with cages made of poly(L-lactide-co-D,L-lactide)/polyglycolic acid (PLDLLA/PGA), with faster results depending on the graft used—in this study, faster with BMP-2. Favorable results for bioabsorbable devices were also found in the study by Cao et al,^28^ in which, after 3 months of follow-up, fusion rates were higher compared with tricortical bone graft and PEEK devices. Similar results were reported by Ren et al,^47^ with higher fusion rates in cervical arthrodesis using bioabsorbable devices compared with titanium and bone devices at 6 months of follow-up.

This study also showed that range of motion (ROM) outcomes were superior when using interbody bioabsorbable devices up to the first 4 months of follow-up compared with traditional techniques, with a pooled OR of 1.70 in biomechanical analyses. In the study by Cao et al.^28^ at 3 months of follow-up, bioabsorbable devices showed significantly lower ROM compared with controls, indicating greater stability of the fused segment. Conversely, Kandziora et al.^20^ did not find improvements in this parameter at 3 months of postoperative follow-up for cervical arthrodesis in sheep, compared with tricortical bone.

Regarding intervertebral disc height, Cao et al^28^ observed a significant increase in sheep treated with bioabsorbable devices compared with isolated bone grafts, with similar heights to those treated with PEEK devices at 3 months. This result was also found in the study by Ren et al.^47^ in which, at 3 and 6 months of cervical arthrodesis in sheep, bioabsorbable cages showed significantly greater final intervertebral height compared with titanium and bone devices. In our analysis, however, no significant differences in disc height parameters were observed over the follow-up period of the included studies. This suggests that bioabsorbable cages may be effective in promoting bone fusion, as differences in vertebral height are not clinically relevant in the short term, while fulfilling their primary role of providing mechanical support to achieve appropriate segmental fusion in conjunction with graft material. These findings suggest that, in shorter periods, the bone response to fusion may be sufficient to preserve intervertebral height.^48^

There are several nuances regarding the use of bioabsorbable interbody devices. In the study by Toth et al.^45^ it was discussed that the benefits of these devices may be limited by factors such as material quality and resorption rate, as resorption over time may affect efficacy due to different local and clinical conditions. Similar findings were reported in studies by Bostman et al.^49,50^ and Cahill et al^51^ highlighting that the clinical effectiveness of bioabsorbable cages depends on several factors, including biocompatibility, resorption rate, and the experimental model used.

In this context, the comparison between bioabsorbable cages and conventional techniques, as observed in the study by Wuisman et al.^38^ indicates that the advantage of biological cages becomes apparent mainly after an adaptation period, which varies depending on the follow-up duration across studies.

A possible explanation for the better outcomes observed with bioabsorbable devices in the early postoperative months lies in the fact that, during this initial period of bone consolidation, implants with properties closer to human bone may carry a lower risk of iatrogenic injuries such as endplate or pedicle fractures—factors that could compromise consolidation. In later stages of fusion, implants with characteristics more similar to host bone reduce the risk of mechanical load deviation caused by more rigid implants (stress shielding), which can result in subsidence and fixation failure.^52^

Furthermore, the low heterogeneity observed among the selected studies reinforces the consistency of the results, suggesting that the techniques used in the included studies were sufficiently homogeneous to support general conclusions. As Thompson e Higgins^35^ emphasize, low heterogeneity in meta-analyses increases confidence in the derived conclusions, especially when parameters are well defined and data are consistent across studies.

The results of this meta-analysis suggest that bioabsorbable interbody devices, by not showing inferiority to conventional treatments in interbody spinal arthrodesis in animals, represent a potential material for incorporation into surgical spine care. These devices offer the theoretical appeal of lower complication rates compared with currently used implants, while fulfilling their primary role of mechanical support, gradually degrading over time, enabling high-quality imaging follow-up, and promoting bone fusion when combined with graft material.

Conventional techniques remain the treatment of choice for cervical spine pathologies requiring intervertebral fusion; however, further studies with longer follow-up are needed to more clearly define the advantages of bioabsorbable interbody devices in the clinical context of human pathology.

Study limitations include variability in cage composition, short animal follow-up, and, although animal studies remain a cornerstone of preclinical research, their limitations require careful interpretation of findings. Complementary approaches, including computational modeling, in vitro experiments, and early-phase clinical trials, are essential to bridge the gap between preclinical and clinical contexts.

## CONCLUSION

The present review showed that bioabsorbable devices, when compared with traditional techniques, demonstrated slightly superior performance during observation periods shorter than four months and, in periods longer than four months, did not show inferiority in outcomes regarding intervertebral fusion in animal studies.
